# Supplementary material for: Co-Targeting ErbB Receptors and the PI3K/AKT Axis in Androgen-Independent Taxane-Sensitive and Taxane-Resistant Human Prostate Cancer Cells
Source: Cancers (Basel). 2022 Sep 23;14(19):4626. doi: 10.3390/cancers14194626 (PMC9561990; doi:10.3390/cancers14194626)
Supplement: Supplementary file 1 [file cancers-14-04626-s001.zip › cancers-1862790-supplementary.pdf]

Supplementary Materials:

Supplementary Table S1. Cell cycle analysis after 24 h treatment with the respective drugs (summary from three independent experiments)

Cell	Treat	G1 (%)	S (%)	G2/M (%)
**PC3**	DMSO	61.12 ± 3.22	11.27 ± 0.60	24.13 ± 1.28
	MK2206	77.49 ± 3.23	4.37 ± 0.18	16.24 ± 0.68
	Lapatinib	67.18 ± 2.57	9.31± 0.36	20.21± 0.77
	MK+Lap	75.54 ± 4.74	1.73 ± 0.11	21.53 ± 1.35
**PC3/Pac**	DMSO	58.78 ± 3.48	16.06 ± 0.96	20.72 ± 1.24
	MK2206	84.37 ± 4.52	3.67 ± 0.20	10.11 ± 0.54
	Lapatinib	69.20 ± 3.24	10.17 ± 0.48	17.34 ± 0.82
	MK+Lap	78.05 ± 6.16	4.16 ± 0.39	15.75 ± 1.25

Cell	Treat	G1 (%)	S (%)	G2/M (%)
**DU145**	DMSO	53.29 ± 4.06	6.72 ± 0.52	36.42 ± 2.80
	MK2206	53.29 ± 3.42	8.13 ± 1.07	34.26 ± 2.20
	Lapatinib	59.45 ± 4.22	6.12 ± 0.60	31.37 ± 2.33
	MK+Lap	63.13 ± 3.69	5.15 ± 0.34	28.77 ± 1.68
**DU145/Doc**	DMSO	59.61 ± 4.38	15.98 ± 4.20	20.15 ± 0.84
	MK2206	64.63 ± 1.58	13.20 ± 2.52	17.30 ± 2.64
	Lapatinib	61.06 ± 1.41	9.59 ± 3.98	24.29 ± 3.50
	MK+Lap	70.80 ± 1.70	8.25 ± 0.90	16.63 ± 1.73

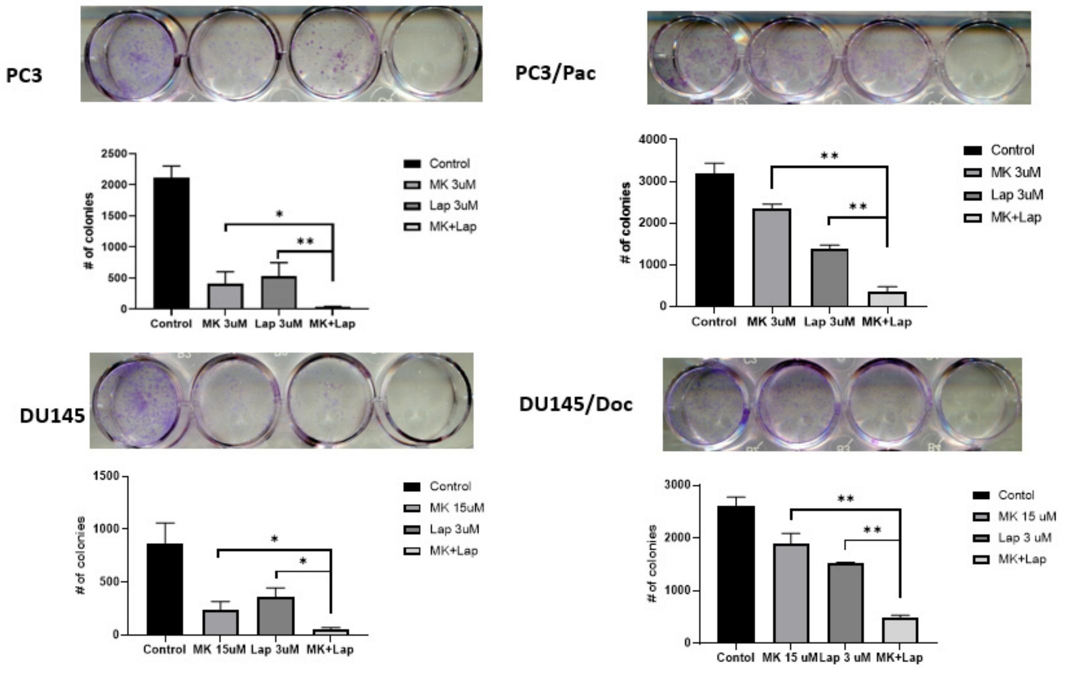

Supplementary Figure S1. 2D Clonogenic assays. Cells seeded in 12-well plates in triplicates were treated with the respective drugs as shown for 10 days. The colonies were then stained with crystal violet, photo-graphed and quantified using ImageJ software. Experiments were repeated three independent times. PC3, PC3/Pac cells were treated with DMSO, lapatinib 3 μM, MK2206 6 μM, or both. DU145, DU145/Doc cells were treated with DMSO, lapatinib 3 μM, MK2206 15 μM, or both. * p-value < 0.05. ** p < 0.01.

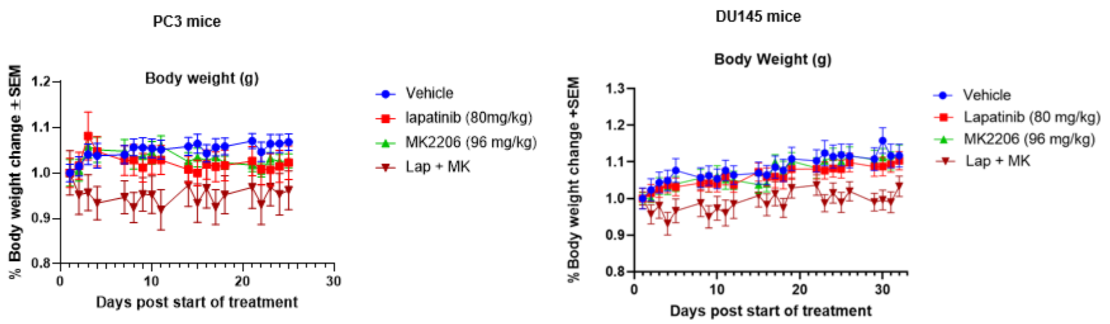

Supplementary Figure S2. Total mouse body weights (in grams) for the different treatment groups assessed M-F of each week.

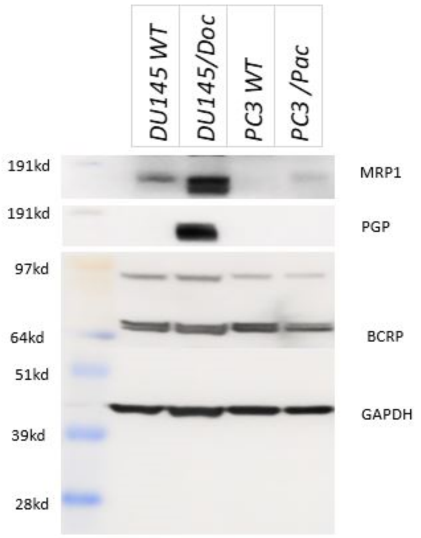

Supplementary Figure S3. Original western blot for Figure 1A showing the bands with molecular weight markers.

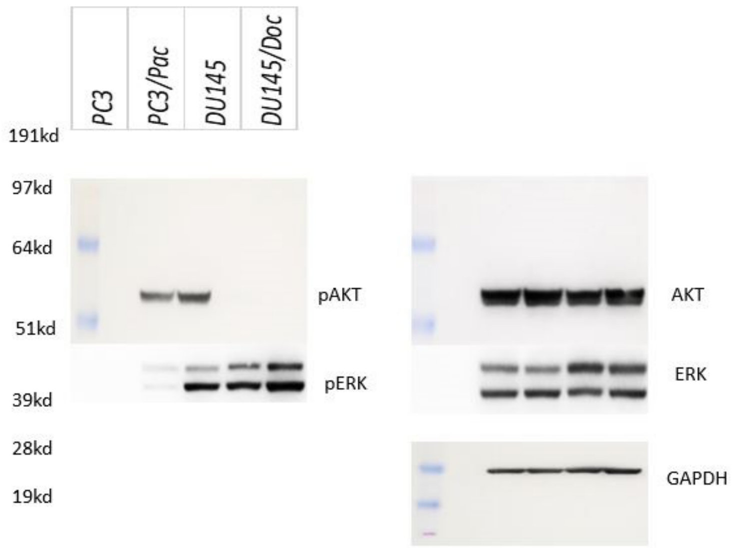

Supplementary Figure S4. Original western blot for Figure 2A showing the bands with molecular weight markers.

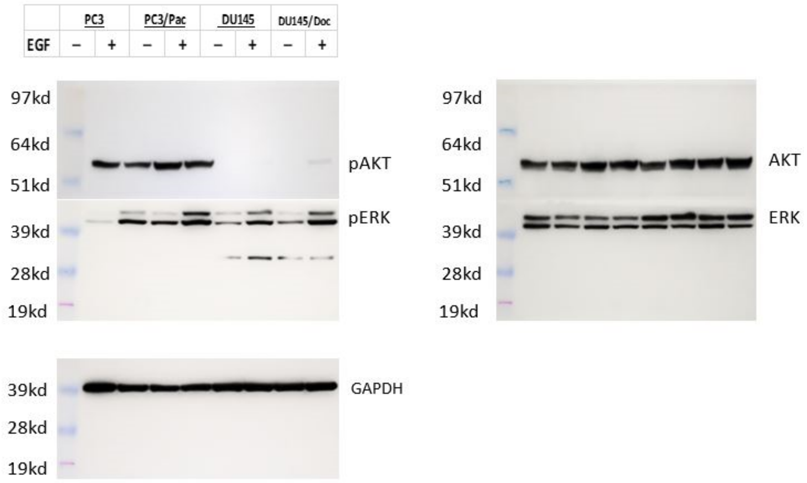

Supplementary Figure S5. Original western blot for Figure 2B showing the bands with molecular weight markers

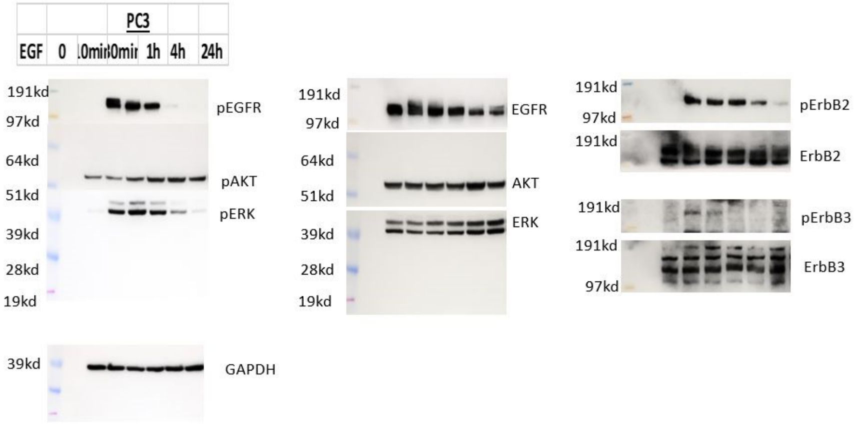

Supplementary Figure S6. Original western blots for Figure 2C showing the bands with molecular weight markers (PC3 cells)

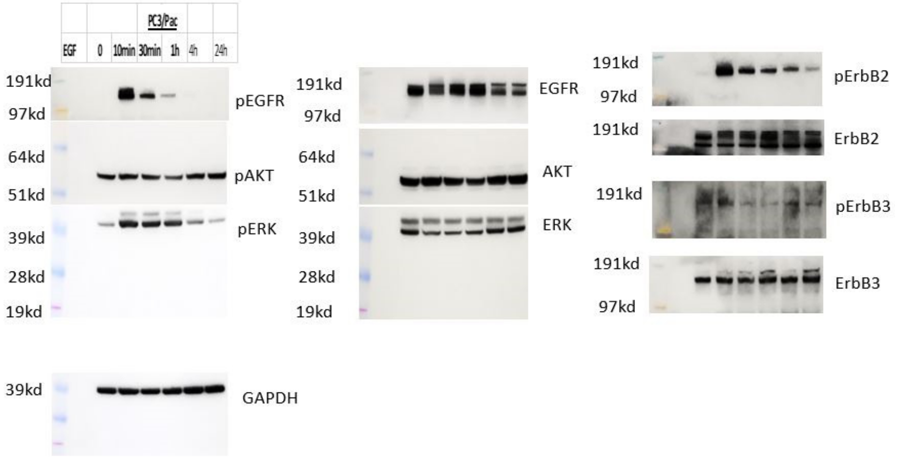

Supplementary Figure S7. Original western blots for Figure 2C showing the bands with molecular weight markers (PC3/Pac cells)

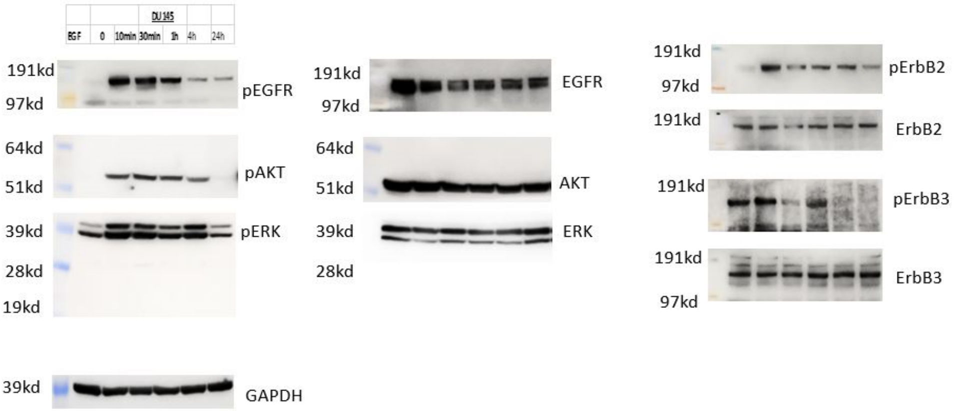

Supplementary Figure S8. Original western blots for Figure 2D showing the bands with molecular weight markers (DU145 cells)

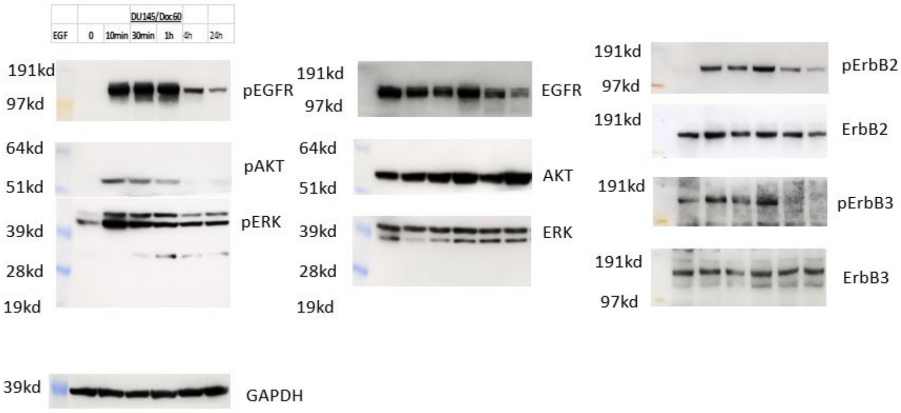

Supplementary Figure S9. Original western blots for Figure 2D showing the bands with molecular weight markers (DU145/Doc cells)

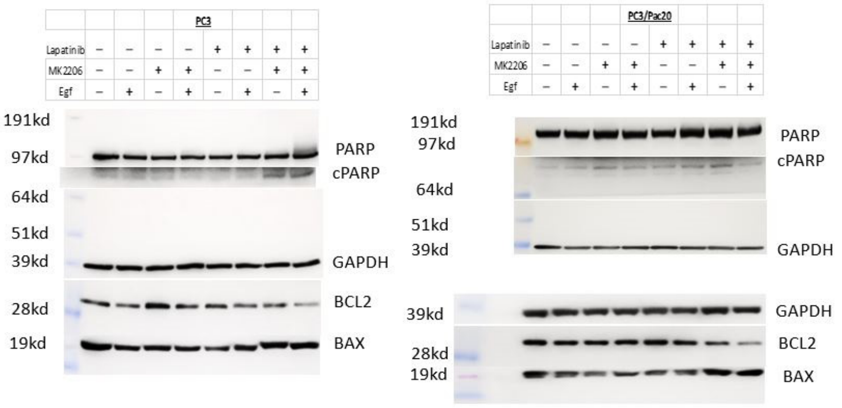

Supplementary Figure S10. Original western blots for Figure 3F showing the bands with molecular weight markers (PC3 and PC3/Pac cells)

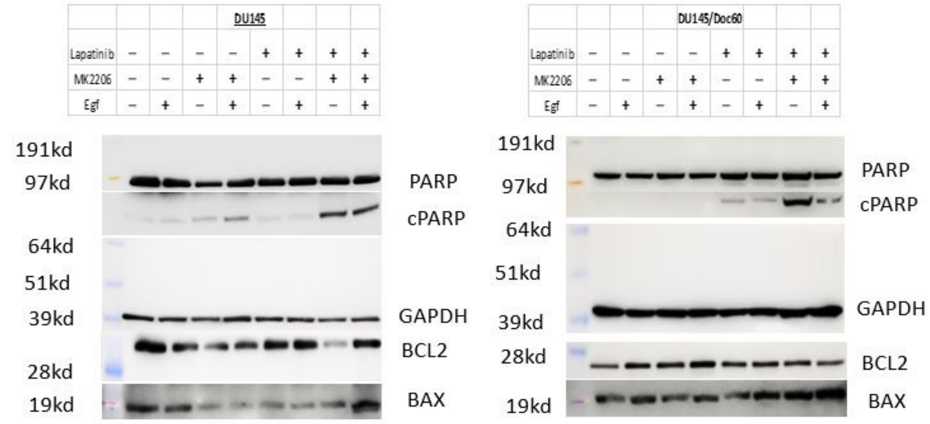

Supplementary Figure S11. Original western blots for Figure 3G showing the bands with molecular weight markers (DU145 and DU145/Doc cells)

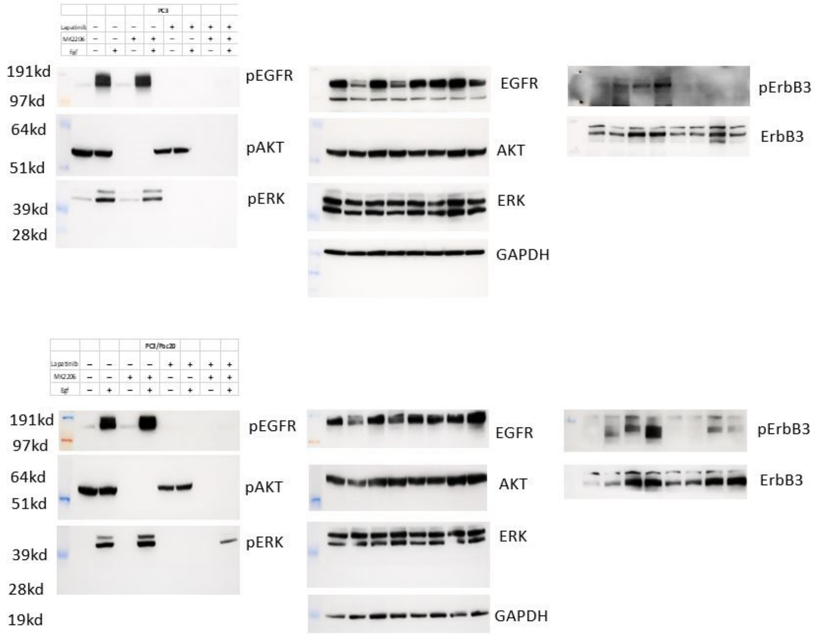

Supplementary Figure S12. Original western blots in Figure 4A, B showing the bands with molecular weight markers (PC3 and PC3/Pac cells)

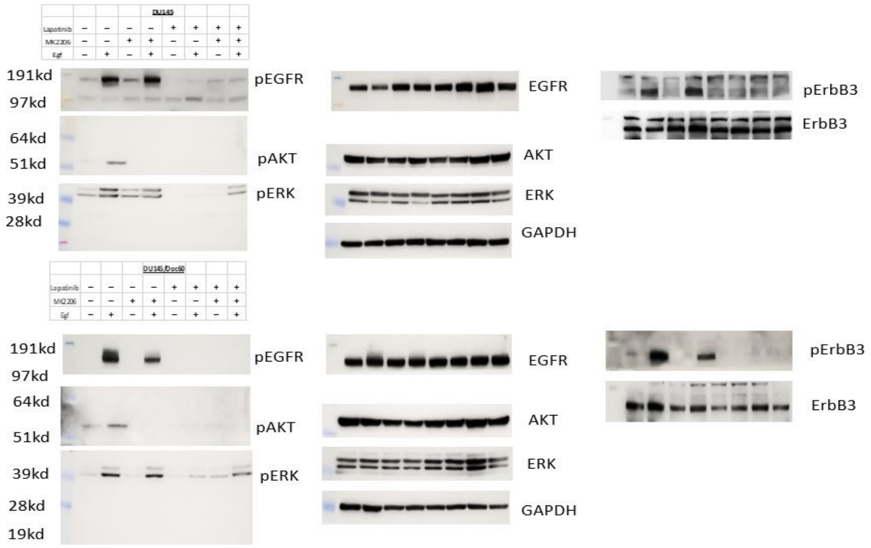

Supplementary Figure S13. Original western blots in Figure 5A, B showing the bands with molecular weight markers (DU145 and DU145/Doc cells)

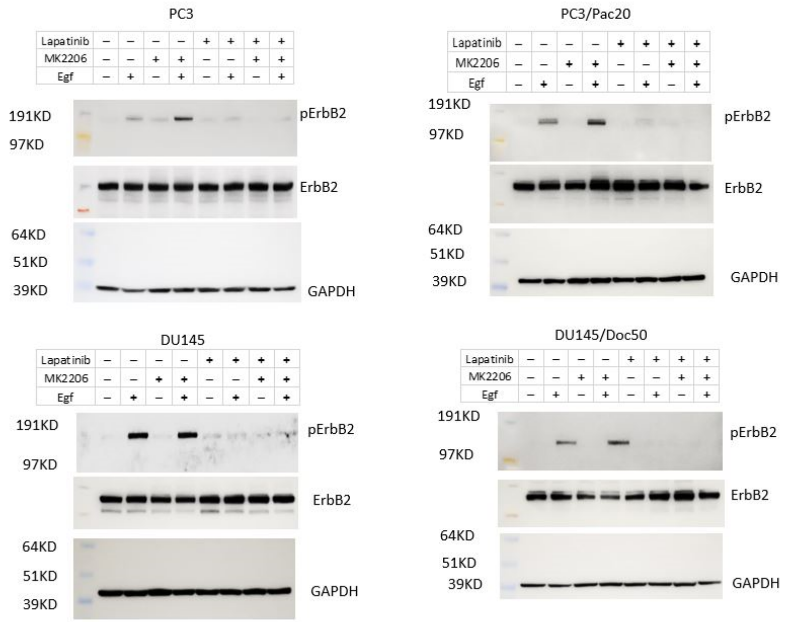

Supplementary Figure S14. Original western blots in Figure 4A, B (PC3, PC3/Pac) and Figure 5A, B (DU145, DU145/Doc) showing the bands with molecular weight markers

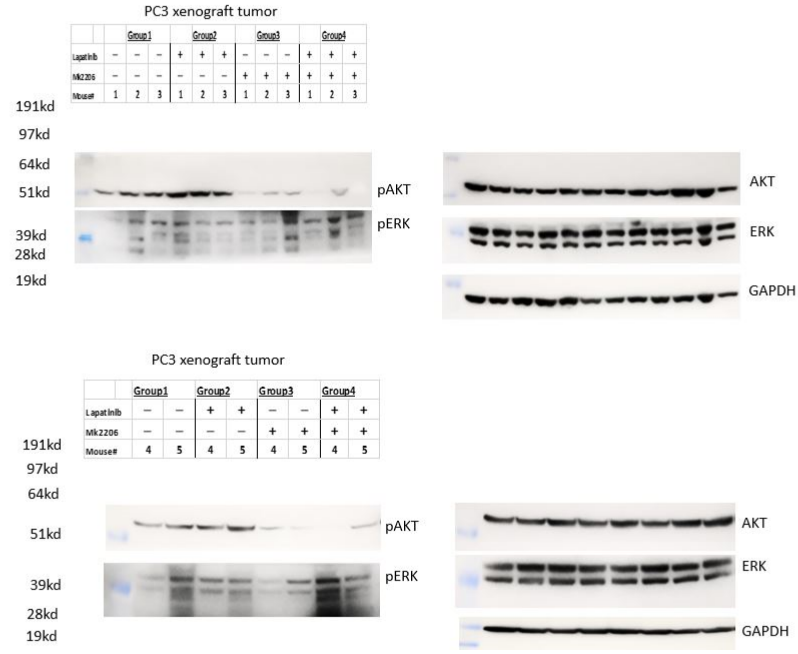

Supplementary Figure S15. Original western blots in Figure 6E showing the bands with molecular weight markers (PC3 xenograft tumors)

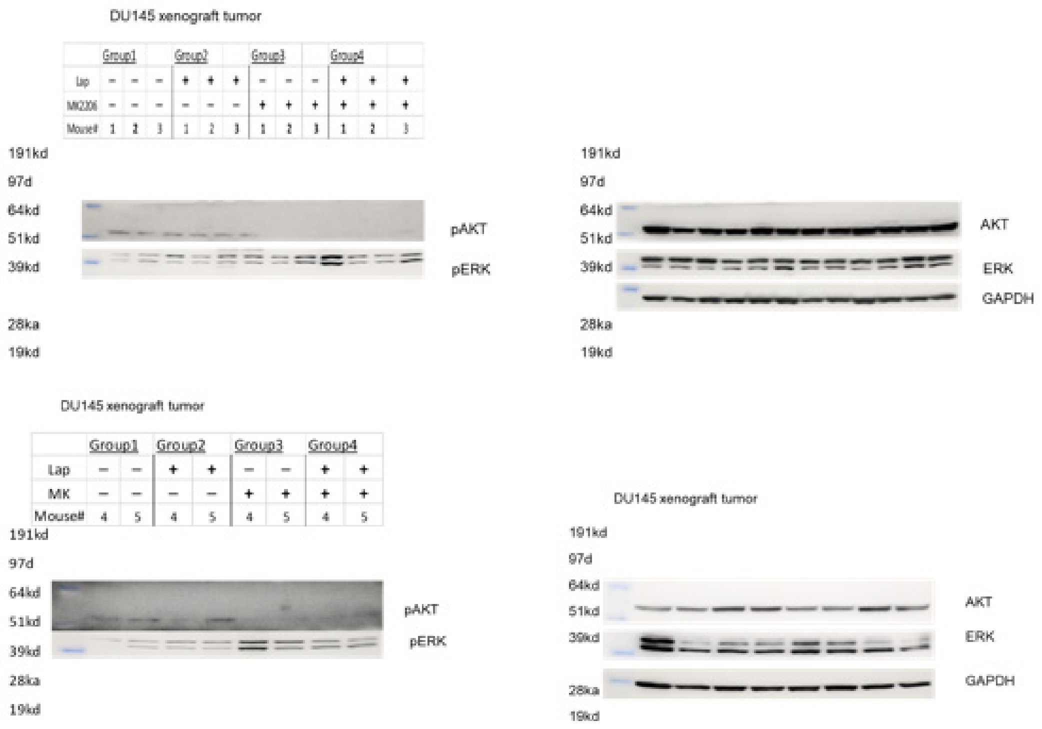

Supplementary Figure S16. Original western blots in Figure 6F showing the bands with molecular weight markers (DU145 xenograft tumors).

Supplementary Table S2. Cell Line authentication report for PC3

Allele Table

Locus	PC3	ATCC Reference PC-3Prostate Adenocarcinoma Human (CRL-1435)
Amelogenin	X	X
CSF1PO	11	11
D13S317	11	11
D16S539	11	11
D21S11	29,31.2	No Data
D5S818	13	13
D7S820	8,11	8,11
TH01	6,7	6,7
TPOX	8,9	8,9
vWA	17	17
Mouse Marker	**Negative**	

Interpretation

The sample provided exhibits identical genetic profiles.

Sample PC3 shares 12 alleles of 12 alleles (100.0 %) with the ATCC references.

The submitted profile is an exact match for the following ATCC human cell line(s) in the ATCC STR database (8 core loci plus Amelogenin): ATCC Reference PC-3 Prostate Adenocarcinoma Human (CRL-1435).

Supplementary Table S3. Cell Line authentication report for DU145

Allele Table

**Locus**	**DU145**	**ATCC Reference DU 145Prostate Carcinoma Human (HTB-81)**
Amelogenin	X,Y	X,Y
CSF1PO	10,11	10,11
D13S317	12,13,14	12,13,14
D16S539	11,13	11,13
D21S11	30,33	No Data
D5S818	10,13	10,13
D7S820	7,10,11	7,10,11
TH01	7	7
TPOX	11	11
vWA	17,18,19	17,18,19
Mouse Marker	Negative	

Interpretation

The sample provided exhibits identical genetic profiles.

Sample DU145 shares 19 alleles of 19 alleles (100.0 %) with the ATCC reference.

The submitted profile is an exact match for the following ATCC human cell line(s) in the ATCC STR database (8 core loci plus Amelogenin): ATCC Reference DU 145Prostate Carcinoma Human (HTB-81).

Supplementary Table S4. Primary and secondary antibody list, company, catalog number and dilutions

Antibody	Company Name	Catalog Number	Dilution
**Primary**			
**pAKT**	Cell Signaling	9271	1:1000
**AKT**	Cell Signaling	92712	1:1000
**pERK**	Cell Signaling	9106	1:2000
**ERK**	Cell Signaling	9102	1:1000
**pEGFR**	Cell Signaling	2235	1:1000
**EGFR**	Cell Signaling	2232	1:1000
**pErbB2**	Cell Signaling	2247	1:1000
**ErbB2**	Proteintech	18299-1-AP	1:1000
**pErbB3**	Cell Signaling	4791	1:1000
**ErbB3**	Cell Signaling	4754	1:1000
**PARP/Cleaved PARP**	Cell Signaling	9542	1:1000
**BCL2**	Cell Signaling	15071	1:1000
**BAX**	Cell Signaling	2772	1:1000
**PGP**	Sigma-Aldrich	P7965	1:1000
**BCRP**	Sigma-Aldrich	B7185	1:1000
**MRP1**	Santa Cruz	18835	1:1000
**GAPDH**	Cell Signaling	2118	1:2000
**Secondary**			
**Anti-mouse**	SeraCare	5450-0011	1:10,000
**Anti-rabbit**	SeraCare	5450-0010	1:10,000
